# A hitchhiker's guide to diffusion tensor imaging

**DOI:** 10.3389/fnins.2013.00031

**Published:** 2013-03-12

**Authors:** José M. Soares, Paulo Marques, Victor Alves, Nuno Sousa

**Affiliations:** ^1^Life and Health Science Research Institute (ICVS), School of Health Sciences, University of MinhoBraga, Portugal; ^2^ICVS/3B's - PT Government Associate LaboratoryBraga/Guimarães, Portugal; ^3^Department of Informatics, University of MinhoBraga, Portugal

**Keywords:** diffusion tensor imaging, hitchhiker's guide, acquisition, analysis, processing

## Abstract

Diffusion Tensor Imaging (DTI) studies are increasingly popular among clinicians and researchers as they provide unique insights into brain network connectivity. However, in order to optimize the use of DTI, several technical and methodological aspects must be factored in. These include decisions on: acquisition protocol, artifact handling, data quality control, reconstruction algorithm, and visualization approaches, and quantitative analysis methodology. Furthermore, the researcher and/or clinician also needs to take into account and decide on the most suited software tool(s) for each stage of the DTI analysis pipeline. Herein, we provide a straightforward hitchhiker's guide, covering all of the workflow's major stages. Ultimately, this guide will help newcomers navigate the most critical roadblocks in the analysis and further encourage the use of DTI.

## Introduction

Diffusion-Weighted Imaging (DWI) (Le Bihan and Breton, 1985; Merboldt et al., 1985; Taylor and Bushell, 1985; Le Bihan et al., 1986) is a variant of conventional Magnetic Resonance Imaging based on the tissue water diffusion rate. It is a non-invasive method, with unparalleled sensitivity to water movements within the architecture of the tissues that uses existing MRI technology and requires no new equipment, contrast agents, or chemical tracers. The introduction of the diffusion tensor model enabled the indirect measurement of the degree of anisotropy and structural orientation that characterizes diffusion tensor imaging (DTI) (Basser et al., 1994a,b; Pierpaoli et al., 1996). While DWI refers to the contrast of the acquired images, DTI is a specific type of modeling of the DWI datasets. DTI principles and basic concepts have been extensively described and reviewed in the literature (Mori and Barker, 1999; Le Bihan et al., 2001; Hagmann et al., 2006; Mori and Zhang, 2006; Mori, 2007; Nucifora et al., 2007; Assaf and Pasternak, 2008; Jones, 2008, 2010b; Mukherjee et al., 2008a; Johansen-Berg and Behrens, 2009; Figueiredo et al., 2011; Thomason and Thompson, 2011; Tournier et al., 2011; Yang et al., 2011). Summarily, the basic concept behind DTI is that water molecules diffuse differently along the tissues depending on its type, integrity, architecture, and presence of barriers, giving information about its orientation and quantitative anisotropy (Chenevert et al., 1990; Moseley et al., 1990; Douek et al., 1991; Beaulieu, 2002). With DTI analysis it is possible to infer, in each voxel, properties such as the molecular diffusion rate [Mean Diffusivity (MD) or Apparent Diffusion Coefficient (ADC)], the directional preference of diffusion [Fractional Anisotropy (FA)], the axial (diffusion rate along the main axis of diffusion), and radial (rate of diffusion in the transverse direction) diffusivity. Diffusion in White Matter (WM) is less restricted along the axon and tends to be anisotropic (directionally-dependent) whereas in Gray Matter (GM) is usually less anisotropic and in the Cerebrospinal fluid (CSF) is unrestricted in all directions (isotropic) (Pierpaoli et al., 1996; Song et al., 2002; Hagmann et al., 2006). Based on this assumption, Basser and colleagues (1994a,b) modeled the diffusion process by an ellipsoid, which can mathematically be represented by a 3 × 3 symmetric matrix, also known as tensor (hence DTI's name origin).

**Figure 1:**
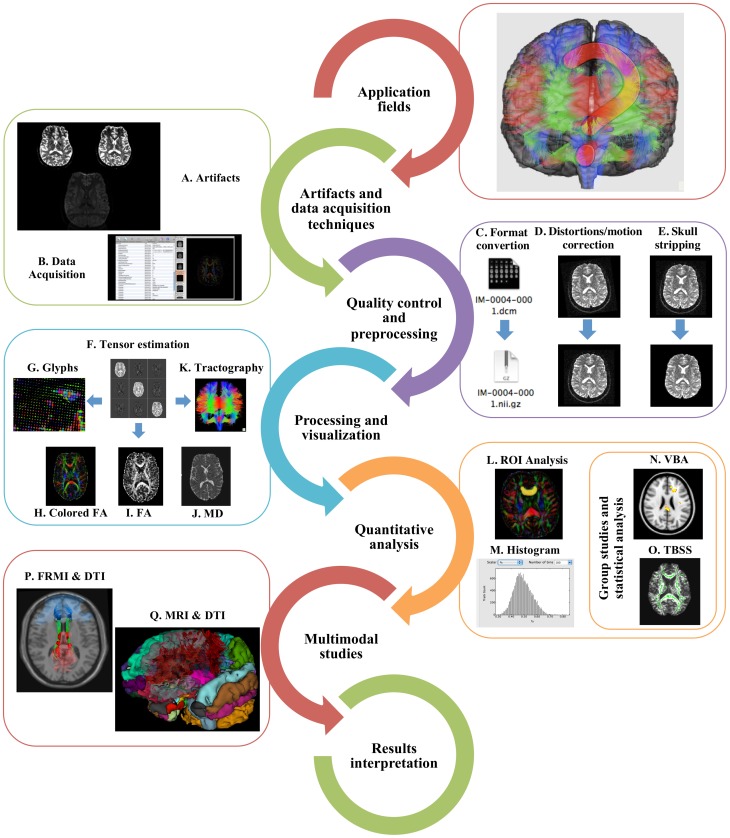
**Typical DTI workflow.** In order to perform a DTI study, researchers need to understand its main application fields, recognize the main artifacts **(A)** and what acquisition protocols can be used **(B)**. The data should undergo quality control, preprocessing, including format conversion **(C)**, distortions and motion correction **(D)**, and skull stripping **(E)**. Before further analysis, tensors need to be estimated **(F)** and the resulting data can be visualized as glyphs **(G)**, scalar indices such as colored FA **(H)**, FA **(I)**, and MD **(J)** or as tractography **(K)**. ROI **(L)**, histogram **(M)**, VBA **(N)**, or TBSS **(O)** analyses may be performed and the results can be incorporated with fMRI **(P)** or structural MRI **(Q)** in multimodal analysis. Finally, results interpretation should be made with extreme caution.

Gaining increased popularity among clinicians and researchers, DTI is presently a promising tool for studying WM architecture in living humans, both in healthy conditions and in disease. However, it has a complex workflow (summarized in Figure 1) that implicates knowledge of imaging artifacts, complex MRI protocol definition, neuroanatomical complexity, and intrinsic technique limitations. These factors are compounded by a multitude of preprocessing and analysis methods in several software packages. Several papers and books describing the main technical issues and pitfalls related to DTI studies have been published (Basser and Jones, 2002; Moritani et al., 2005; Le Bihan et al., 2006; Mukherjee et al., 2008b; Bammer et al., 2009; Jones, 2010a,b,c; Jones and Cercignani, 2010; Chung et al., 2011; Hasan et al., 2011) which are complemented by available neuroanatomical WM atlas (Jellison et al., 2004; Wakana et al., 2004; Catani and Thiebaut De Schotten, 2008; Lawes et al., 2008; Oishi et al., 2008, 2010; Zhang et al., 2010; Bazin et al., 2011; Nowinski et al., 2012). However, given the constant methodological advances and the increase in DTI applicability across clinical and research domains, we have here compiled a practical hitchhiker's guide of critical information and main references to consider in setting up DTI studies, optimizing data quality, and interpreting results.

## Application fields

DTI is sensitive to microstructural tissue properties and, thus, it can be used in WM research and clinical work to explore WM anatomy and structure *in vivo*. In fact, this sensitivity, providing diffusion summary measures and tissue fiber orientation, has made DTI widely used as a clinical tool, especially in conditions where abnormalities in WM are expected (Sundgren et al., 2004; Mori and Zhang, 2006). For example, it has been successfully implemented to study patients with acute stroke or brain tumors; neurodegenerative disorders including multiple sclerosis, epilepsy, and Alzheimer's; neuropsychiatric disorders such as schizophrenia; mild cognitive impairment; development disorders like dyslexia, autism, and attention deficit hyperactivity disorder; movement disorders (mainly Parkinson's and Huntington's); neurogenetic developmental disorders such as Williams syndrome and fragile X syndrome; and changes in WM microstructure during neurodevelopment and in aging (Le Bihan et al., 2001; Moseley et al., 2002; Sundgren et al., 2004; Vilanova et al., 2006; Nucifora et al., 2007; Ciccarelli et al., 2008; Imfeld et al., 2009; Johansen-Berg and Behrens, 2009; Madden et al., 2009; Yamada et al., 2009; Chanraud et al., 2010; Carvalho Rangel et al., 2011; Fung et al., 2011; Hygino da Cruz Jr et al., 2011; Thomason and Thompson, 2011; Voineskos et al., 2012). DTI variables (e.g., FA, axial diffusivity) are usually related with alterations in structure (possibly due to particular conditions/disease) pointing to specific myelination levels and axonal injury (Song et al., 2002; White et al., 2008; Budde et al., 2009; Gupta et al., 2012). With the progressive increase in the range of applications, consistency of results and robustness of the technique, DTI is expected to be valuable in the future in disease treatment planning, detection of preclinical markers, and microstructural abnormalities; it is also anticipated that the structural-functional correlates provided by DTI studies will become part of the clinic's imaging routine.

## Artifacts and data acquisition techniques

Implementing DTI studies involves the understanding of specific MRI acquisition techniques and artifacts, and how to deal with them (Figures 1A,B). The artifacts in DWI datasets are mainly related with the gradient system hardware, pulse sequence, acquisition strategy used and motion. DWI data are generally collected to cover the entire brain by repeating the acquisition while varying the orientation or magnitude of the diffusion gradients. DWI has low Signal-to-Noise Ratio (SNR) and resolution and is very susceptible to motion (Farrell et al., 2007; Choi et al., 2011; Polders et al., 2011). To reduce the influence of motion artifacts, the scan time can be reduced. This makes the use of Single-shot Echo Planar Imaging (EPI) (Mansfield, 1977; Stehling et al., 1991; Turner et al., 1991; Nana et al., 2008) the typical strategy employed to reduce this sensitivity (Stehling et al., 1991; Turner et al., 1991; Nana et al., 2008); however, alternative sequences, such as Fast Spin Echo (FSE) (Seifert et al., 2000; Pipe et al., 2002), Line Scan Diffusion Imaging (LSDI), (Gudbjartsson et al., 1996) and Stimulated Echo Acquisition Mode (STEAM) (Nolte et al., 2000) may also be of interest to reduce artifacts (Xu et al., 2004; Mukherjee et al., 2008b; Bammer et al., 2009).

Besides being the most common approach, EPI images are very sensitive to other artifacts related with EPI characteristics such as field inhomogeneities at B0 (especially at higher fields), image blurring [also called Point-Spread Function (PSF) artifact], limited resolution from T2 and T2^*^ signal decay during the signal readout; with diffusion MRI properties such as eddy current-induced distortions and general MRI issues like sensitivity to motion and B0-susceptibility artifacts (Farzaneh et al., 1990; Basser and Jones, 2002; Le Bihan et al., 2006; Kaur et al., 2007; Bammer et al., 2009; Jones and Cercignani, 2010). Shorter readout times can reduce the echo train and increase SNR, resulting in a lower sensitivity to motion and reduced susceptibility to geometric artifacts and blurring (Mukherjee et al., 2008b). This decrease in the readout times can be achieved with the use of phased-array head coils, enabling parallel imaging such as Sensitivity Encoding (SENSE), Array Spatial Sensitivity Encoding Technique (ASSET), and Generalized Autocalibrating Partially Parallel Acquisition (GRAPPA) (Pruessmann et al., 1999; Bammer et al., 2002; Griswold et al., 2002; Jaermann et al., 2006; Brau et al., 2008; Nana et al., 2008; Holdsworth et al., 2009). Parallel imaging is important at 3T, and essential at 7T (Mukherjee et al., 2008c).

Importantly, the two main artifacts intrinsic to DTI acquisitions that may destroy the voxel-wise correspondence across all the DWIs are eddy current distortions and head motion (Rohde et al., 2004; Le Bihan et al., 2006; Mohammadi et al., 2010). In DTI, contrary to most imaging acquisitions, the gradients are much longer (rising and falling edges of the gradient are separated in time); there might be perturbations of the local magnetic field that result in current inductions in the diverse conducting surfaces of the MRI scanner causing image distortions (contraction and/or overall shift and shear) that are usually easy to detect visually. Eddy currents vary with the diffusion gradient applied and, consequently, there will be misregistration between successive images, which are worse with stronger and longer gradient pulses. Some strategies have been used to prevent and correct eddy current distortions, based on a twice-refocused spin echo pulse, bipolar gradients, field maps, and preprocessing approaches (described later); it is important to note, however, that there are pitfalls associated with these strategies (Reese et al., 2003; Chen et al., 2006; Zhuang et al., 2006; Huang et al., 2008; Truong et al., 2008, 2011; Jones and Cercignani, 2010).

Diffusion MRI is very sensitive to motion, due to phase shifts induced microscopically by diffusion-driven water molecular displacements, and macroscopically by head motion, cardiac pulsation and breathing. This sensitivity increases with the intensity and duration of gradient pulses, which are characterized by the *b*-value, the scalar that defines the amount of diffusion weighting in the experiment (Le Bihan et al., 2001). It can be reduced by synchronizing the acquisition with the source of motion, monitoring using “navigator echoes,” using specific protocols, applying real-time prospective motion and outlier detection methods; however, all of these may raise other problems such as increased acquisition times (Ordidge et al., 1994; Pipe, 1999; Kennedy and Zhong, 2004; Zwiers, 2010; Zhou et al., 2011b; Kober et al., 2012; Ling et al., 2012). Even though it is also possible, and even advisable, to correct subject motion using preprocessing techniques (see in preprocessing steps below), the best approach is still to use comfortable padding to adjust the participant's head, and to inform the subject in advance about the noise and the vibration of the bed. This vibration was recently reported as the cause of another artifact, known as vibration artifact. During the acquisition, strong gradients are applied causing low-frequency mechanical resonances of the MR system that lead to small brain tissue movements. When these movements occur in the direction of the diffusion-encoding gradient, phase offsets will occur inducing signal dropouts in DWI images. This kind of artifacts can be reduced increasing TR (with the drawback of reducing SNR) or using full k-space coverage combined with parallel imaging (e.g., GRAPPA) (Gallichan et al., 2010). It can also be compensated using methods such as phase-encoding reversal (COVIPER) (Mohammadi et al., 2012), implemented in *Artifact Correction in Diffusion MRI* (ACID) toolbox.

Whenever artifacts can't be corrected, such as severe movement, signal dropouts, or slice-wise intensity disruption, researchers adopt different strategies depending on the type and extent of the artifact. The exclusion of the affected subject, volume (gradient) or single slice is a common approach. An alternative is to limit the analysis to regions without artifacts if the artifact is localized (Liu et al., 2010b).

Artifacts in DWI acquisitions lead to errors in tensor estimation and, consequently, in diffusion maps (FA and MD) that give rise to fiber reconstructions with erroneous orientation or length. Optimizing diffusion-imaging sequences is, thus, crucial to obtain more precise data. Protocols should be oriented to the question under study, and specific parameters should be used to optimize a particular analysis. There is no consensus on the optimal acquisition parameters because they vary according to MRI hardware configuration, field strength, vendor, scanning time available, specific anatomic structure and brain anatomic coverage needed. Thus, herein we only provide a suggestion for parameters in a typical DTI acquisition mostly based on a previous technical review (Mukherjee et al., 2008b). Usually, DWI data are acquired covering the entire brain through axial slices with no gap between slices (crucial for tractography). On modern scanners, 5 min scanning time is enough to perform an acceptable acquisition; however, the acquisition time may be much longer (15 min) depending on the scanner and the acquisition parameters defined. Diffusion tensor estimation requires high *b*-values (e.g., 1000 s/mm^2^) along at least six non-collinear diffusion encoding directions in addition to one minimally T2 weighted low *b*-image (*b* = 0 s/mm^2^). Several sampling schemes have been suggested and it is argued that the sampling vectors should be uniformly distributed in space so that the SNR is also uniform in respect to the tensor orientation. The usage of 30 diffusion-encoded images (orientations) was found to be a good compromise between image quality and scanning time, since increasing the number of orientations didn't result in improved tensor orientation and MD estimates (Jones, 2004). Ideally, 1 low-b image for each 5–10 high-b images should be acquired. Another approach is to repeat acquisition of the same DWIs [increasing the Number of Excitations (NEX)] instead of raising the number of DWIs (Wang et al., 2011). Most DTI studies use high *b*-values in the range of 700–1000 s/mm^2^, and the actual standard for clinical DWI is 1000 s/mm^2^ (Mukherjee et al., 2008b). The magnitude of *b*-values is also dependent upon SNR, echo time, eddy currents, and motion artifacts and should, in specific cases, be adjusted to the population and specific structure. The rule is that the optimal *b*-value multiplied by the ADC value should be close to 1 (Xing et al., 1997; Jones et al., 1999a). The spatial resolution is also important for DTI quality and when using isotropic voxels (in-plane resolution and thickness with equal dimensions, e.g., 2 × 2 × 2); typically, 2–2.5 mm are recommended for fiber tracking, using interleaved acquisitions to minimize crosstalk between contiguous sections. Anisotropic voxels also introduce bias in the quantitative assessment of fiber orientation and anisotropy and larger voxels are more likely to have more than one fiber tract orientation (Mukherjee et al., 2008b). Other characteristic parameters of DTI acquisitions are Field Of View (FOV) usually ranging from 240 to 256 mm, acquisition matrix 96 × 96–128 × 128, Echo Time (TE) 50–70 ms and Repetition Time (TR) 8.5–12 s. Optimization of DTI protocols has been the focus of diverse studies that specify metrics for detailed protocol definition, including its relation with the DTI metrics and multi-center approaches (Xing et al., 1997; Jones et al., 1999a; Pfefferbaum et al., 2003; Hagmann et al., 2006; Farrell et al., 2007; Wakana et al., 2007; Mukherjee et al., 2008b; Abe et al., 2010; Jones, 2010c; Lagana et al., 2010; Choi et al., 2011; Hasan et al., 2011; Zhu et al., 2011; Lebel et al., 2012).

## Quality control and preprocessing

Quality control and preprocessing procedures are key steps to detect and correct artifacts in DWI and to exclude those that could not be corrected, providing consistency to reliable tensor estimation. Although it is already possible to find automated preprocessing pipelines (Liu et al., 2010b) (ColbyImaging, http://www.colbyimaging.com/wiki/neuroimaging/dti-preprocessing), there is no consensus over which workflow is ideal for DTI quality control or preprocessing. Herein we provide a guided approach, comprising standard methodology easy to perform and not extremely time consuming.

The first step consists in, when importing the data, checking if all images have been imported and sorted correctly and if the different subjects, under the same study, have the same parameters. This can be performed with general-purpose image viewers such as *Osirix*, *syngo FastView*, *MRIcro*, or *ImageJ* (Rosset et al., 2004; Liao et al., 2008). After this initial examination, a visual inspection of the DWI data is recommended to detect potential artifacts. Looping through the raw images in different “orthogonal” views allows the identification of geometric distortions, signal dropouts, subtle system drifts, and missing slices (Tournier et al., 2011). On the other hand, outlier detection methods provide automated approaches to identify corrupted images. Methods based on testing for ADC consistency (Jiang et al., 2009) and detection of spike noise (Chavez et al., 2009) have also been suggested. *RESTORE* is a commonly used tool to estimate tensors robustly, excluding potential outliers prior to tensor estimation (Chang et al., 2005). Monte Carlo simulated data have been used to study the effects of different sources/magnitudes of noise on DTI derived measures (Pierpaoli et al., 1996; Basu et al., 2006) and also to validate methods such as RESTORE.

The acquired data may at this point need several preprocessing steps depending on the MRI scanner, acquisition parameters, image quality, software package used and study focus. In preprocessing, it is common to start by converting raw data into specific and adequate image formats (Figure 1C). With poor interoperability between DTI analysis tools and the lack of a standard DTI format (Patel et al., 2010), many software packages define their own data formats; for example, Neuroimaging Informatics Technology Initiative (NIfTI) and Analyze and Nearly Raw Raster Data (NRRD) are common data formats. File format converters such as *MRIcro*, *dcm2nii*, *MRIConvert, NiBabel*, and software package converters (e.g., *AFNI*, *Freesurfer*, *SPM*, *Slicer*) are commonly used to convert from the original DICOM format (Smith et al., 2004; Pieper et al., 2006; Friston et al., 2007; Fischl, 2012).

In DWI images, distortions caused by eddy currents and head motion are the most common artifacts (Figure 1D); therefore, a common and recommended preprocessing step is to correct for such artifacts. Eddy currents can be corrected with an affine registration to the b0 image and motion correction with a rigid body registration to b0. Since both corrections consist in registration procedures, they can be implemented as one single step. To do this, *FMRIB's Diffusion Toolbox* (FDT), *Automated Image Registration* (AIR), and *DT_Recon*, are popular software tools, although tools like *DTIC* (b0 and eddy current correction for DTI) *DTIPrep* (Liu et al., 2010b), *DIFF_PREP*, and *ExploreDTI* (Leemans et al., 2009) can also be used for this purpose. It is important to note that since this procedure deals with changes in the orientation of the images, the encoding vectors should be reoriented (Leemans and Jones, 2009); fortunately, the *DTIPrep*, *DIFF_PREP*, and *ExploreDTI* tools take this into account.

After this, one optional step is to perform skull stripping (Figure 1E), removing non-brain areas from analysis, improving co-registration/normalization results and reducing data size. This step can be accomplished with several tools, such as *BET* from FSL, *Freesurfer*, *Atropos*, and *Bioimage Suite*. Accurate tensor estimation and tractography analysis are also dependent on precise gradient tables. Gradient information can usually be retrieved directly from the MRI console or it can be calculated with specific tools such as *DTI gradient table creator*. In some cases, the orientation of the gradient directions may be inaccurate and minor corrections may be required (ColbyImaging, http://www.colbyimaging.com/wiki/neuroimaging/bvecs). After tensor estimation (described in the following sections), visual examination of tensor orientations in some specific regions (e.g., corpus callosum, cingulate and uncinate fasciculus) is also an assessment that can be performed with any tensor visualization tool (e.g., *Slicer*, *TrackVis*, *DTIStudio*, *MedINRIA*, *BrainVoyager QX*, *FSL View*, *Camino*, *BioImage Suite*, *ExploreDTI*). If tensor orientation errors are noticed it is necessary to modify the gradient table and repeat the tensor reconstruction (using tools such as *DTI-TK*). The presence of bias in DTI datasets is also common (Farrell et al., 2007) and it can originate from multiple sources (e.g., noise, field inhomogeneities, quality control procedures that might modify/exclude problematic gradients, and experimental and biological parameters); at this stage, it can be estimated with the SIMulation and EXtrapolation (SIMEX) statistical approach (Lauzon et al., 2011).

On a final practical note, researchers or clinicians can search and compare existing software in listings such as the *source for neuroimaging tools and resources* (NITRC, http://www.nitrc.org/) or *I Do Imaging* (http://www.idoimaging.com/), particularly when searching for a tool for a specific task.

## Processing and visualization

After data preprocessing, the next stage in DTI analysis comprises tensor estimation at each voxel (Figure 1F); for this purpose, images with diffusion-encoding gradients applied at least along six non-collinear directions are required. Three main methods are used to estimate the tensors: Ordinary Least Squares (OLS), the most popular, and Weighted Linear Least Squares (WLLS) and Non-linear Least Squares (NLLS). Different estimation methods may yield different results, therefore it is important to assure that the same package is used to estimate the tensors in an entire dataset (Koay et al., 2006; Jones and Cercignani, 2010). Since the diffusion tensor is a symmetric 3 × 3 matrix, it can be described by its eigenvalues (λ1, λ2, λ3) and eigenvectors (*e*1, *e*2, *e*3). The eigenvalues and eigenvectors are then used to process scalar indices and, in some studies, tractography analysis. At each voxel, the eigenvalues represent the magnitude of diffusion and the corresponding eigenvectors reflect the directions of maximal and minimal diffusion.

Nowadays, a wide range of free or commercial DTI software tools with different purposes and specifications are available. Choosing one among the others can be a difficult and time-consuming task for newcomers. In Table 1 we present a list of the most commonly used software tools and their main applicability.

**Table 1:** **Software tools for DTI processing used in published studies**.

**DTI software/tools**	**URL**	**Main purpose**
*3D Slicer* (Pieper et al., 2006)	http://www.slicer.org/	Tensor estimation, ROI analysis, and tractography
*AFNI* (Cox, 2012)	http://afni.nimh.nih.gov/afni	Preprocessing and tensor estimation
*BioImage Suite* (Papademetris et al., 2005)	http://www.bioimagesuite.org/	Tensor estimation, ROI analysis, and tractography
*BrainVoyager QX* (Goebel, 2012)	http://www.brainvoyager.com/	Tensor estimation and tractography
*Camino* (Cook et al., 2006)	http://web4.cs.ucl.ac.uk/research/medic/camino/pmwiki/pmwiki.php?	Tensor estimation and tractography
*Dipy* (Garyfallidis et al., 2011)	http://dipy.org	Tensor estimation and tractography
*DoDTI* (Park et al., 2004)	http://neuroimage.yonsei.ac.kr/dodti/	Preprocessing, tensor estimation, and tractography
*DTI-Query* (Akers et al., 2004)	http://graphics.stanford.edu/projects/dti/software/	Tractography
*DTI-TK* (Zhang et al., 2009)	http://dti-tk.sourceforge.net/pmwiki/pmwiki.php	Registration
*DTIStudio* (Jiang et al., 2006)	https://www.mristudio.org/wiki/DtiStudioV2	Tensor estimation, ROI analysis, and tractography
*ExploreDTI* (Leemans et al., 2009)	http://www.exploredti.com/	Preprocessing, tensor estimation, and tractography
*Freesurfer* (Fischl, 2012)	http://surfer.nmr.mgh.harvard.edu/	Preprocessing and tensor estimation
*FSL-FDT* (Smith et al., 2004)	http://www.fmrib.ox.ac.uk/fsl/fdt/index.html	Preprocessing, tensor estimation, and tractography
*FSL-TBSS* (Smith et al., 2006)	http://www.fmrib.ox.ac.uk/fsl/tbss/index.html	TBSS analysis
*JIST* (Lucas et al., 2010)	http://www.nitrc.org/projects/jist/	Preprocessing and tensor estimation
*MedINRIA* (Toussaint et al., 2007)	http://wwwsop.inria.fr/asclepios/software/MedINRIA/	Tensor estimation, tractography, and ROI analysis
*MrDiffusion*	http://white.stanford.edu/mrdiff	Tensor estimation and tractography
*MRtrix* (Tournier et al., 2012)	http://www.brain.org.au/software/mrtrix/	Tensor estimation and tractography
*SATURN* (Cardenes et al., 2010)	http://www.lpi.tel.uva.es/saturn/	Tensor estimation and tractography
*SPM and toolboxes* (e.g., *Diffusion II, DTI Toolboxes*)	http://www.fil.ion.ucl.ac.uk/spm/ext/	Preprocessing and tensor estimation
*TrackVis* (Wang et al., 2007)	http://trackvis.org/	Tensor estimation, tractography, and ROI analysis
*TORTOISE* (Pierpaoli et al., 2010)	https://science.nichd.nih.gov/confluence/display/nihpd/TORTOISE	Preprocessing, tensor estimation, and ROI analysis

Mainly used for clinical purposes, commercial applications although intuitive, friendly and automated, are also more rigid and limited. Examples of these are *syngo DTI*, *Elite Neuro clinical solutions*, or *Functool and FiberTrak* and others such as *nordicICE Diffusion/DTI Module, iPlan® Fibertracking, Prism Clinical Imaging®, DynaSuite Neuro*. On the other hand, Python based tools, especially the Nipy project (including tools such as *Dipy*, *NiBabel*, and *Nipype*) are more flexible and customizable, but less intuitive and user friendly (Millman and Brett, 2007).

One of the biggest challenges in DTI is to visualize and present the tensor information in an intuitive and easily understandable way. In fact, the high dimensionality of the data and the complex associations in diffusion tensors domain make this step quite problematic. Typical approaches consist of using tensor glyphs or reducing the dimensionality to one scalar (scalar indices) and to three dimensions (tractography). Tools such as *Explore DTI*, *MedINRIA, and Slicer*, among many others, enable all the before mentioned visualization schemes, as summarized in Table 2.

**Table 2:**
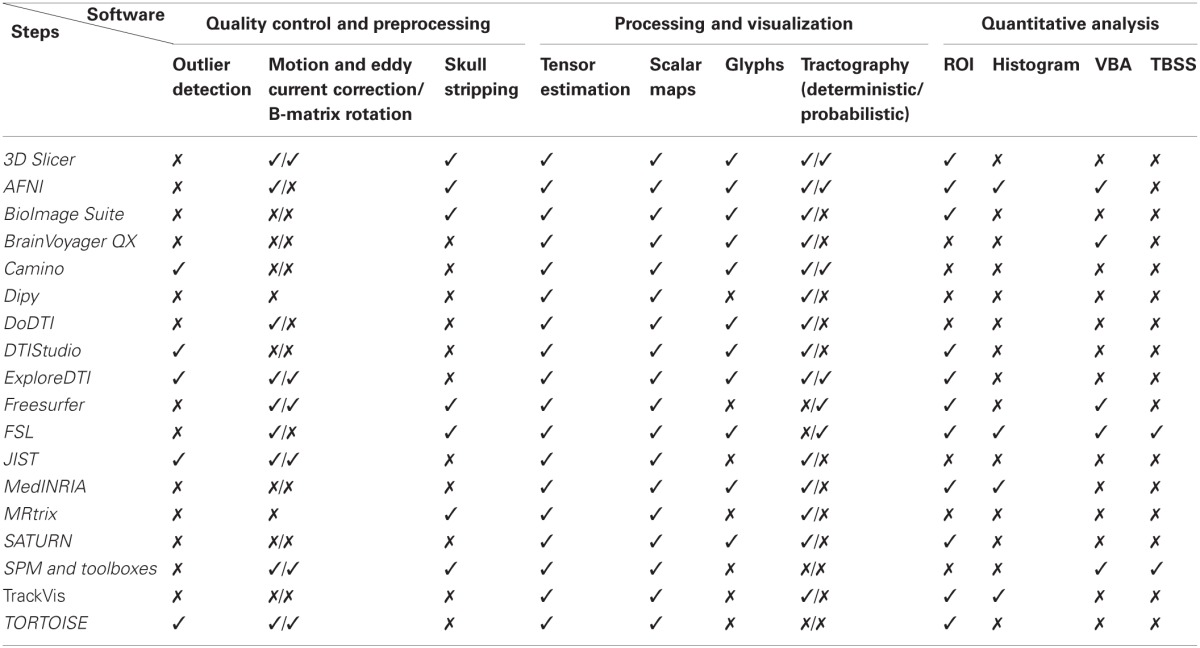
A list of the main workflow steps implemented by the common DTI tools^*^.

* To the best of our knowledge at the date of submission, based on information gathered from the software manuals, main webpages, and published papers.

2D visualization of scalar maps, the most common DTI visualization approach used by clinicians, is used due to its simplicity and instant visualization; however, this approach has limitations in the quantity of information presented. The two main diffusion indices, MD and FA, are based on the eigenvalues, which represent the magnitude of the diffusion process.

MD, ADC, or trace, can be calculated by the mean of the three eigenvalues and correspond to the molecular diffusion rate (lower values mean low diffusivity) (Figure 1J):

$$MD=\frac{\lambda_{1}+\lambda_{2}+\lambda_{3}}{3}=\frac{D_{xx}+D_{yy}+D_{zz}}{3}=\frac{\text{Trace}}{3}$$

where *D*_*xx*_, *D*_*yy*_, *D*_*zz*_ are the diagonal terms of the diffusion tensor.

Fractional Anisotropy is a normalized measure of the fraction of the tensor's magnitude due to anisotropic diffusion, corresponding to the degree of anisotropic diffusion or directionality and ranges from 0 (isotropic diffusion) to 1 (anisotropic diffusion):

$$FA=\sqrt{\frac{3}{2}}\sqrt{\frac{{\left(\lambda_{1}-D\right)}^{2}+{\left(\lambda_{2}-D\right)}^{2}+{\left(\lambda_{3}-D\right)}^{2}}{\lambda_{1}^{2}+\lambda_{2}^{2}+\lambda_{3}^{2}}}$$

where *D* = (λ_1_ + λ_2_ + λ_3_)/3. FA has no information about the orientation (Figure 1I), its rotationally invariant. This can be deciphered by color-coded FA maps in which the color of each voxel demonstrates its main diffusion direction (Figure 1H). In these maps, red color represents left-to-right orientation, green posterior-to-anterior and blue inferior-to-superior diffusion. Other relevant DTI indices reported are trace (magnitude of diffusion in a voxel), Lattice Anisotropy Index (LAI—an intervoxel anisotropy measure with reduced sensitivity to noise), axial (derived from the largest eigenvalues and measures the rate of diffusion in the direction of fastest diffusion detecting longitudinal diffusion along axons), and radial diffusivity (derived from the second and third eigenvalues and measures the transverse direction of diffusion) (Basser and Pierpaoli, 1996; Vilanova et al., 2006; Jones, 2008; Abe et al., 2010; Chanraud et al., 2010). Typically, MD is higher in damaged tissues as a result of increased free diffusion; in contrast, FA decreases due to the loss of coherence in the main preferred diffusion direction. Importantly, software tools presented in Table 1 that can be used for tensor estimation also enable the calculation of some of the most commonly used scalar maps.

Contrasting with the dimensionality reduction of the tensor represented by scalar indices, glyphs are parameterized graphical objects that describe a diffusion tensor through its size, shape, location, and color (Figure 1G). Glyphs are used for visualization and quality control and not for analysis procedures. The most typical representation is the 3D ellipsoidal shape elongated along the fastest diffusion axis and squashed along restricted diffusion directions. These objects map the tensor eigenvectors and eigenvalues, which express the water molecules diffusion profile (Pierpaoli and Basser, 1996; Kindlmann and Westin, 2006). Other shapes can also be used, such as box glyphs which are better for linear anisotropy profiles but, as ellipsoids, they overlook important information and it is difficult to clearly understand 3D shapes when viewed in planes (Vilanova et al., 2006). To overcome this problem, another class of glyphs, known as superquadrics were introduced and combine spherical, cylindrical and box shapes to distinguish between isotropic, planar and linear anisotropy and intermediate states (Kindlmann, 2006). The main disadvantage of glyph visualization is that it only allows intrinsic individual profiles rather than global characterization of the tensor data. Most DTI viewers enable glyphs representation as lines, tubes or ellipsoids and allow its combination with scalar maps visualization. From the tools presented in Table 2, *MedINRIA* and *SATURN* also support box and superquadrics glyphs.

The last family of parameters that can be extrapolated from DTI is based on the primary eigenvector of diffusion to obtain three-dimensional representations of WM pathways or fiber bundles, the so-called, WM tractography (Figure 1K). This method projects 3D trajectories of fiber pathways and connection patterns between different brain systems *in vivo* (Jones et al., 1999b; Mori et al., 1999; Basser et al., 2000; Mori and van Zijl, 2002; Wedeen et al., 2012). Tractography processing can be divided in three main stages, namely seeding, propagation, and termination. Seeding consists of defining the points from which the fiber bundles will be drawn; one of the most common methodologies is based on defining Regions Of Interest (ROIs) and placing one or more seeds in each voxel of the ROI (Figure 1L). The ROIs can be manually drawn or extracted from other MRI modalities. The main issues at this stage are related with the location of the seeding points among different subjects and the fiber tracking tool used, causing variability in the results (Burgel et al., 2009; Hattingen et al., 2009). A second popular approach consists of using automatic seeding for the whole brain, enabling a fully exploratory visualization of the tensor data.

During the propagation process the fibers are gradually generated. Fiber tracking can be performed with different algorithms divided in two main categories: deterministic and probabilistic (Jones, 2008, 2010a; Descoteaux et al., 2009; Chung et al., 2011; Fillard et al., 2011; Tensaouti et al., 2011; Tournier et al., 2011). Deterministic tractography aims to model the data and, in practical terms, can be thought of as generating/reconstructing one fiber from each seed. On the other hand, probabilistic approaches take into account the uncertainty of the estimation, which results in probability maps representing the likelihood of a voxel being part of a fiber and provides the multiple possible fiber directions emanating from each seed. A common deterministic algorithm used and implemented in the main DTI processing packages is Fiber Assignment by Continuous Tracking (FACT) defining specific anatomic tracts based on ROIs assuming that fiber orientation is uniform within a voxel and changes abruptly in the boundaries of it (Mori et al., 1999). Other deterministic algorithms are streamlining with different interpolation methods (tri-linear, second, or fourth order Runge-Kutta), tensor deflection, or tensorline (Weinstein et al., 1999; Basser et al., 2000; Lazar et al., 2003). Tools like *Diffusion Toolkit* implement all these algorithms, whereas *Slicer* implements second order Runge-Kutta. Regularly used probabilistic algorithms are PICo (Parker et al., 2003), used by *Camino*, multi-fiber field model (Behrens et al., 2007), implemented in *FSL*, and the Bayesian approach (Friman et al., 2006) used in *Slicer* and *Camino*.

The last tractography step is termination of the fiber tracking procedure based on some well-defined criteria, also known as the termination criteria. These criteria aim to avoid propagating the fibers in voxels where robustness of the vectorial field is not assured. The common termination criteria are minimum FA thresholds (typically 0.1–0.3 in adult brain and 0.1 in infant) and turning angle threshold (typically 40–70°, depending on the pathway).

Interpreting tractography maps can be problematic due to the intrinsic unrealistic assumption of a homogeneous unidirectional population inside the voxels. Specific regions of the brain containing two or even more differently oriented fiber bundles within the same voxel (crossing, diverging, or kissing fibers) lead to incorrect estimations of fiber directions and pathways and abrupt terminations of tracts (Wiegell et al., 2000; Alexander et al., 2001; Barrick and Clark, 2004; Descoteaux et al., 2009). This limitation can be minimized by adopting more sophisticated approaches including multi tensor models, High Angular Resolution Diffusion Imaging (HARDI), Hybrid Diffusion Imaging (HYDI), Diffusion Spectrum Imaging (DSI), Q-Ball Imaging (QBI), Q-Space Imaging (QSI), Spherical Deconvolution Model, and Persistent Angular Structure MRI (PAS-MRI) (Tuch et al., 2002; Jansons and Alexander, 2003; Tuch, 2004; Alexander et al., 2006; Wedeen et al., 2008; Assemlal et al., 2011; Tournier et al., 2011; Landman et al., 2012; Vos et al., 2012). Recently, these methods have gained increasing popularity, replacing the traditional tensor model for tractography (Wedeen et al., 2012). For instance, DSI and QBI use probability density functions instead of single tensors, which can describe the diffusion process in many different directions at each voxel. This comes with the limitation of requiring longer acquisition times as it needs more encoding directions (Tournier et al., 2011). HARDI, DSI and QBI approaches can be implemented with *TrackVis* and *Diffusion Toolkit*, and *Camino* has been used in HYDI analysis and PAS-MRI.

## Quantitative analysis

After parametric maps (e.g., MD, FA) computation and in order to perform individual or group statistical analysis, the next common step is to extract summary measures from either specific anatomical regions or whole brain. For this purpose, ROIs, histogram, voxel-based analysis and Tract-Based Spatial Statistics (TBSS) are typically applied. It is important to note that usually researchers/clinicians are interested in group comparisons and the methods to extract summary measures differ mainly in the way the correspondence across subjects is achieved.

ROI analysis is based on manual delineation of *a priori* specific regions of the brain or on automated parcellations. ROI analyses are time-consuming, require anatomical knowledge and are applied to quantify diffusion parameters (mainly MD and FA) within those areas. The main problems of ROI analyses include: the influence of the image intensity on ROI boundaries by direct segmentations on the map of interest (typically FA or MD); the difficulty to co-register diffusion with typical anatomical images (T1 or T2 weighted) when using anatomical ROIs; performing analysis in smaller/thinner tracts; and difficult application in longitudinal studies (Snook et al., 2007; Mukherjee et al., 2008b; Astrakas and Argyropoulou, 2010; Chanraud et al., 2010; Jones and Cercignani, 2010). Of note, ROI analysis can be performed with the main tensor estimation and visualization software, such as *Slicer*, *TrackVis*, *MedINRIA*, and *ExploreDTI*.

Another possibility for quantitative analysis is the use of frequencies of distributions to screen the voxels within a specific range of parameters of interest (usually MD or FA). The histogram of each diffusion parameter presents the mean, the peak height and location, values that can be used to compare groups through statistical tests (Figure 1M). Histograms allow analysis of whole brain in an automated way, without any *a priori* specified ROI; however, such an approach requires the removal of the tissue of no interest (typically CSF), does not retain any information about the location of abnormalities and is sensitive to partial volume effect from atrophy (Della Nave et al., 2007; Jones and Cercignani, 2010; Zhou et al., 2011a). For such approach, tools such as *TrackVis* or *MedINRIA* can be used.

Analyses on a voxel-by-voxel basis are becoming popular in DTI given that they are automated, require minimum intervention and are not influenced by users. Voxel Based Analysis (VBA) involves registration of diffusion maps into a standard space (a process known as normalization) to achieve correspondences between subjects across voxels and consequently anatomical structures (Figure 1N). This enables the comparison of diffusion parameters between groups and correlations with covariates of interest (e.g., age). This approach allows spatially specific (as ROIs) and unbiased (as histogram) analysis and does not require previous ROI definition. The main problem is the accuracy of registration algorithms using tensor datasets (Mukherjee et al., 2008b; Abe et al., 2010; Astrakas and Argyropoulou, 2010; Jones and Cercignani, 2010; Van Hecke et al., 2010). VBA can be carried out with SPM or *BrainVoyager QX*, with SPM as the most widely used software tool for this kind of analysis.

A recent method designed to overcome the problems with registration algorithms and arbitrariness of spatial smoothing is TBSS. TBSS is an automated method for detecting group voxel-wise changes in whole brain, based on the skeletonization of group registered FA maps (Figure 1O). TBSS removes the need to perform spatial smoothing, increases the statistical power (reducing number of total voxels tested). On the other hand, the skeletonization of FA images may be inaccurate in images with large anatomical shifts or WM lesions and registration errors are difficult to identify visually in the skeleton (Jones and Cercignani, 2010). Back projection to native space is also an issue since the skeletonization process aligns local maxima, which may not necessarily correspond to the same anatomical location across all subjects (Zalesky, 2011). This method is part of the *FSL* distribution (Smith et al., 2006).

One of the big issues in group studies is that to compare a condition among a group, the individual images need to be normalized to a standard space (Evans et al., 2012). After this, each structure should be in the same position across all the group subjects. The normalization procedure is crucial for VBA analysis and the result of a misalignment can be unpredictable. This is particularly challenging in DTI due to its highly directional and topographical nature. This challenge led to a variety of procedures for the normalization of DTI images (Jones et al., 2002; Park et al., 2003; Xu et al., 2003). The most straightforward method consists in using the b0 images to calculate a rigid alignment with a high-resolution T1 image and then an affine alignment from the T1 space to standard the space. Note that the transformation matrixes generated should only be applied to the scalar images. Alternatively, some researchers opt to drive the registration directly from b0 image to an EPI template in standard space. The differences among these approaches were already addressed in previous studies (Liu et al., 2010a). Another normalization approach consists in the normalization of the tensors using complex multi-channel algorithms (Park et al., 2003). The tools that are most commonly used in data normalization are *AIR*, *FLIRT*, and *SPM*. Also here, to solve the normalization problems with DTI images, TBSS provides a new and revolutionary method for inter-subject registration of FA maps.

To perform statistical parametric analysis, the data can be smoothed using a three-dimensional filter. This increases the SNR, reduces imperfections due to spatial normalization procedures, improves the statistical power and enables the assumption of random field theory (Westin et al., 2002). Care must be taken since the chosen spatial width of the filter will determine the size of the differences than can be detected, and smoothing also increases the amount of partial volume effect (Jones and Cercignani, 2010). This step can be performed with tools like *fslmaths* (a command line tool from *FSL* library) or *SPM*.

For a global guidance about the possible software solutions for the main steps of the DTI workflow consult Table 2.

## Multimodal studies

Collecting multimodal brain data from the same individual using different neuroimaging methods has become recently a standard in the field and is certainly a trend for the future. Combining different modalities allows a global and complementary overview of living brain structure and function. Brain connectivity studies have become popular nowadays with the combination of microstructural organization (DTI) and functional activation patterns using resting state or task related functional MRI (fMRI) (Le Bihan, 2012) (Figure 1P). Changes in diffusion measures can point to alterations in functional patterns and behavior. A general problem using this approach is the common need to expand GM clusters to WM areas, in order to reach the WM fiber tracts (Li et al., 2012). In practice this is achieved by dilating the activation clusters using tools such as *fslmaths* (from *FSL* package) or *MarsBaR* (*SPM* toolbox). Several studies have demonstrated neuroanatomical connections between functionally linked brain regions in resting state networks (Van Den Heuvel et al., 2008, 2009; Greicius et al., 2009) and task related patters (Propper et al., 2010; Ethofer et al., 2011).

Another popular multimodal approach is to use conventional MRI or T1 weighted derived masks, segmentations or parcellations (ROIs) in DTI data to extract combined structural and diffusion data (Figure 1Q). Moreover, WM volumetric ROIs have been used to assess microstructure integrity (Fjell et al., 2008; Moriya et al., 2010; Kochunov et al., 2011). In addition, combining DTI parameters with electroencephalography (EEG) records (Bangera et al., 2010; Gullmar et al., 2010), cortico-cortical evoked potentials (CCEPs) (Conner et al., 2011), Magnetoencephalography (MEG) (Fernandez et al., 2011), Positron Emission Tomography (PET) (Yakushev et al., 2011), Magnetic Resonance Spectroscopy (MRS) (Tang et al., 2007; Wang et al., 2009) and Transcranial Magnetic Stimulation (TMS) (Hubers et al., 2012), have revealed new insights on the complementary complexity of the human brain. Importantly, in order to combine DTI with other neuroimaging modality data, the ROIs should be in the same reference space as the DTI data.

## Results interpretation

In DTI data interpretation the most common misconception is related with the scalar results. Usually higher MD and lower FA values indicate damaged or impaired fiber integrity due to increased diffusion and loss of coherence on preferred movement direction. However, this is not always true. In fact, depending on the brain region, cellular basis, and sample studied (specific disease processes, developmental conditions), unusually high or low indices may indicate dysfunction or not (Hoeft et al., 2007; Thomason et al., 2010).

Other pitfalls in DTI interpretation are also observed. The interpretation of color-oriented maps is also far from trivial because two different tracts may have the same color (if they have the same in-plane fiber orientations) and the same tract can change color-coding when orientation changes, making it difficult to localize across 2D images. Crossing fibers are also problematic in DTI results interpretation, affecting more FA, axial and radial diffusivity than MD, and having a huge impact on tractography methods (Tournier et al., 2011).

As a final take-home message, WM integrity assumptions must always be made with extreme caution (Beaulieu, 2002; Jones et al., 2012).

## Conclusions and future directions

DTI is currently a promising tool to study WM microstructure *in vivo*. It has, nevertheless, difficulties associated with each specific analysis stage that must be taken into account from the initial steps of the experimental design to the final interpretation of results. This article has highlighted the common problems faced when performing DTI studies and some possible ways to overcome them, giving practical guidelines and references, including for the most used tools for each step of the common DTI pipeline. The description of the most commonly used solutions and tools in the DTI workflow is something that we believe to be underestimated so far. It is, thus, our belief that this hitchhicker's guide will be of help for newcomers to the field, but also for those who want to update their knowledge on the topic.

## Conflict of interest statement

The authors declare that the research was conducted in the absence of any commercial or financial relationships that could be construed as a potential conflict of interest.
